# Clinicopathologic characteristics of high expression of Bmi-1 in esophageal adenocarcinoma and squamous cell carcinoma

**DOI:** 10.1186/1471-230X-12-146

**Published:** 2012-10-18

**Authors:** Bonnie Choy, Santhoshi Bandla, Yinglin Xia, Dongfeng Tan, Arjun Pennathur, James D Luketich, Tony E Godfrey, Jeffrey H Peters, Jun Sun, Zhongren Zhou

**Affiliations:** 1 Department of Pathology and Laboratory Medicine, University of Rochester Medical Center, 601 Elmwood Ave, Rochester, NY, 14642, USA; 2 Department of Surgery, University of Rochester Medical Center, 601 Elmwood Ave, Rochester, NY, 14642, USA; 3 Department of Biostatistics and Computational Biology, University of Rochester Medical Center, Rochester, NY, 14642, USA; 4 Department of Gastroenterology, University of Rochester Medical Center, Rochester, NY, 14642, USA; 5 Department of Biochemistry, Rush University Medical Center, Cohn Research Building, 1735 W. Harrison St., Room 506, Chicago, IL, 60612, USA; 6 Department of Pathology, University of Texas MD Anderson Cancer Center, 1515 Holcombe Blvd, Houston, TX, 77030, USA; 7 Department of Cardiothoracic Surgery, University of Pittsburgh Medical Center C-800, Presbyterian University Hospital, Pittsburgh, PA, 15213, USA

**Keywords:** Esophageal adenocarcinoma, Bmi-1, Squamous cell carcinoma, Barrett’s esophagus, Dysplasia, High expression, Biomarker, Overall survival

## Abstract

**Background:**

High expression of Bmi-1, a key regulatory component of the polycomb repressive complex-1, has been associated with many solid and hematologic malignancies including esophageal squamous cell carcinoma. However, little is known about the role of Bmi-1 in esophageal adenocarcinoma. The aim of this study is to investigate the amplification and high expression of Bmi-1 and the associated clinicopathologic characteristics in esophageal adenocarcinoma and squamous cell carcinoma.

**Methods:**

The protein expression level of Bmi-1 was detected by immunohistochemistry (IHC) from tissue microarrays (TMA) constructed at the University of Rochester from using tissues accrued between 1997 and 2005. Types of tissues included adenocarcinoma, squamous cell carcinoma and precancerous lesions. Patients’ survival data, demographics, histologic diagnoses and tumor staging data were collected. The intensity (0–3) and percentage of Bmi-1 expression on TMA slides were scored by two pathologists. Genomic DNA from 116 esophageal adenocarcinoma was analyzed for copy number aberrations using Affymetrix SNP 6.0 arrays. Fisher exact tests and Kaplan-Meier methods were used to analyze data.

**Results:**

By IHC, Bmi-1 was focally expressed in the basal layers of almost all esophageal squamous mucosa, which was similar to previous reports in other organs related to stem cells. High Bmi-1 expression significantly increased from squamous epithelium (7%), columnar cell metaplasia (22%), Barrett’s esophagus (22%), to low- (45%) and high-grade dysplasia (43%) and adenocarcinoma (37%). The expression level of Bmi-1 was significantly associated with esophageal adenocarcinoma differentiation. In esophageal adenocarcinoma, Bmi-1 amplification was detected by DNA microarray in a low percentage (3%). However, high Bmi-1 expression did not show an association with overall survival in both esophageal adenocarcinoma and squamous cell carcinoma.

**Conclusions:**

This study demonstrates that high expression Bmi-1 is associated with esophageal adenocarcinoma and precancerous lesions, which implies that Bmi-1 plays an important role in early carcinogenesis in esophageal adenocarcinoma.

## Background

Esophageal carcinoma is the 8th leading cancer in incidence and 6th in mortality worldwide, but it is one of the least studied cancers [1,2]. Squamous cell carcinoma and adenocarcinoma are the two histologic types that make up for greater than 90 percent of the diagnoses of esophageal cancers [3]. Worldwide, the majority of esophageal cancers are squamous cell carcinoma [2]. However, in the United States and Western countries, there has been a dramatic rise in the incidence of esophageal adenocarcinoma to equal or exceed the incidence of esophageal squamous cell carcinoma [4]. Esophageal carcinoma carries a poor prognosis with an overall five-year survival rate of approximately 15 percent in the United States [5]. More than 50 percent of patients have either unresectable tumors or radiographically visible metastases at the time of diagnosis [6]. Identification of early diagnostic markers with high sensitivity and specificity will provide physicians with valuable information for diagnosis, prognosis, and possible treatment options of esophageal carcinoma. Previous studies have suggested the order of events that leads to esophageal adenocarcinoma from normal esophageal epithelium to reflux esophagitis, followed by Barrett’s esophagus, dysplasia, to esophageal adenocarcinoma [7]. During these events, a series of genetic and epigenetic aberrations driven by inflammation and oxidative stress contributes to the carcinogenesis. However, the oncogenetic mechanisms of esophageal adenocarcinoma remain unclear.

The Bmi-1 (B cell-specific Moloney murine leukemia virus integration site 1) gene, a member of the polycomb-group proteins, was first isolated as an oncogene that cooperates with c-myc in the oncogenesis of murine lymphomas [8-10]. It functions as a transcriptional repressor through chromatin modification and plays a role in axial patterning, cell cycle regulation, hematopoiesis, and senescence [11,12]. In addition, deregulation of polycomb-group gene expression leads to cell proliferation and tumor progression [13,14]. Aberrant Bmi-1 expression has been associated with many solid and hematologic malignancies, including mantle cell lymphoma [15], Hodgkin lymphoma [16], B-cell non-Hodgkin lymphoma [17], gastric carcinoma [18], hepatocellular carcinoma [19], colorectal cancer [20,21], breast cancer [22,23], bladder cancer [24], nasopharyngeal carcinoma [25], oral squamous cell carcinoma [26] and non-small cell lung cancer [27]. More recently, studies have reported an association between Bmi-1 expression and esophageal squamous cell carcinoma [28-30]. However, little is known about the role of Bmi-1 in esophageal adenocarcinoma.

The aims of this study are (1) to investigate the association of high Bmi-1 expression with the oncogenic progression of esophageal adenocarcinoma from squamous mucosa, columnar cell metaplasia, Barrett’s esophagus, low- and high-grade dysplasia to adenocarcinoma, and (2) to determine the relationship of high Bmi-1 expression with clinicopathologic characteristics including gender, age, differentiation, and tumor stage in both esophageal adenocarcinoma and squamous cell carcinoma.

## Methods

### Construction of Tissue Microarray

Tissue microarrays, containing 80 cases of squamous epithelium, 63 cases of columnar cell metaplasia, 36 cases of Barrett’s esophagus, 20 cases of low-grade dysplasia, 14 cases of high-grade dysplasia, 110 cases of esophageal adenocarcinoma, and 34 cases of esophageal squamous cell carcinoma, were constructed from representative areas of formalin-fixed specimens collected from 1997 to 2005 in the Department of Pathology and Laboratory Medicine, University of Rochester Medical Center/Strong Memorial Hospital, Rochester, NY. All research was performed under protocols approved at URMC with the title “Biomarkers of esophageal carcinoma” and RSRB case number: RSRB00028546. The 5-μm sections were cut from tissue microarrays and stained with H&E to confirm the presence of the expected tissue histology within each tissue core. Additional sections were cut for immunohistochemistry analysis.

### Pathologic definition of esophageal adenocarcinoma and precancerous lesions

Columnar cell metaplasia was defined as columnar cells without goblet cell metaplasia including mucous glands or mixture of mucous and oxyntic glands. Barrett’s esophagus was defined as mucous glands with goblet cell metaplasia. Low-grade dysplasia was defined as elongated, crowded, hyperchromatic, mucin depletion and pseudo-stratified nuclei with relatively preserved crypt architecture. High-grade dysplasia was defined as marked cytologic abnormality and significant architectural complexity of the glands. Cytologic abnormalities included nuclear pleomorphism, loss of polarity, irregularity of nuclear contour, increased ratio, and increased number of atypical mitoses. Significant architectural complexities of the glands included crypt budding, branching, marked crowding or villiform contour, intraluminal papillae, bridges or a cribriform growth pattern. Esophageal adenocarcinoma was defined as the single cells, small or large irregular glands with both cytologic abnormality and architectural complexity infiltrating into submucosa or deeper layers of esophagus.

### Patients for Tissue Microarrays

All the 110 patients with esophageal adenocarcinoma used for the tissue microarray construction were treated with esophagectomy at University of Rochester Medical Center/Strong Memorial Hospital from 1997 to 2005 without pre-operation neoadjuvant therapy. These patients included 98 males (89%) and 12 females (11%). The patient age ranged from 34 to 85 years with a mean of 65 years (Table 1). The stage, lymph node with or without metastasis, and differentiation information were listed in Table 2. The follow-up period after esophagectomy ranged from 0.03 to 142 months with a mean of 39 months.

**Table 1:** Distribution of patients by histologic types

	**Male**		**Female**	
Histologic type	**n (%)**	**Age (mean)**	**n (%)**	**Age (mean)**
Adenocarcinoma	**98 (89)**	**65**	**12 (11)**	**70**
High-grade dysplasia	**12 (86)**	**65**	**2 (14)**	**71**
Low-grade dysplasia	**20 (100)**	**66**	**0 (0)**	NA*
Barrett’s esophagus	**32 (89)**	**66**	**4 (11)**	**69**
Columnar cell metaplasia	**56 (89)**	**64**	**7 (11)**	**66**
Squamous cell carcinoma	**24 (71)**	**65**	**10 (29)**	**61**
Squamous epithelium	**63 (79)**	**64**	**17 (21)**	**65**

NA- non-available.

**Table 2:** Association of high Bmi-1 expression with age, gender, lymph node metastasis, stage and differentiation in esophageal adenocarcinoma

		**Bmi-1 Non-high expression**	**Bmi-1 High-expression**	***p *value**
**Age**		66 (34–83)	63 (43–85)	**0.284**
**Gender**				**0.529**
	**Male**	60	38	
	**Female**	9	3	
**Lymph node metastasis**				**0.283**
	**Positive**	52	27	
	**Negative**	17	14	
**p Staging**				**0.412**
	**I**	16	12	
	**II**	32	18	
	**III**	10	5	
	**IV**	7	3	
**Differentiation**				**0.004**
	**Well**	44	19	
	**Moderate**	19	15	
	**Poor**	2	4	

### Patients for Affymetrix SNP 6.0 analysis

Frozen tumors were obtained from 116 patients undergoing esophagectomy at the University of Pittsburgh Medical Center, Pittsburgh, PA between 2002 and 2008. The patients' ages ranged from 43 to 88 and the cohort consisted of 95 males and 21 females. The final pathologic stages were stage I (28), stage II (31), stage III (49) and stage IV (7). All tumor specimens were evaluated by a pathologist and determined to be >70% tumor cell representation. Further information on this patient cohort and a comprehensive genomic analysis of these tumors were published by Dulak *et al*. [31]. Microarray data on this cohort has been submitted to the Gene Expression Omnibus (GSE36460) and was made public ( http://www.ncbi.nlm.nih.gov/geo/query/acc.cgi?acc=GSE36460). A research was performed under protocols approved at both participating institutions.

### Affymetrix SNP 6.0 analysis

Genomic DNA was isolated using the QiaAmp DNA Mini Kit (Qiagen, CA), and 600ng was used for labeling and array hybridization at the SUNY Upstate Medical University microarray core facility (Syracuse, NY) using kits and protocols provided by Affymetrix. Array data quality was assessed using Affymetrix Genotyping Console 3.0 and all further data analysis was performed using Nexus 5.0 Copy Number Analysis software (Biodiscovery, Inc. CA).

### Immunohistochemistry

Tissue sections from the tissue microarray were deparaffinized, rehydrated through graded alcohols, and washed with phosphate buffered saline. Antigen retrieval for Bmi-1 was performed by heating sections in 99°C water bath for 40 minutes. After endogenous peroxidase activity was quenched and nonspecific binding was blocked, ready-to-use mouse monoclonal antibody anti-Bmi-1 (Millipore, MA) was incubated at room temperature for 30 minutes. The secondary antibody (Flex HRP) was allowed to incubate for 30 minutes. After washing, sections were incubated with Flex DAB Chromogen for 10 minutes and counterstained with Flex Hematoxylin for 5 minutes. A colon adenocarcinoma with known high Bmi-1 expression served as positive control. Negative control was performed by replacing anti-Bmi-1 antibody with normal serum. A few core samples did not survival from the immunohistochemical staining.

### Scoring of Immunohistochemistry

**Figure 1:**
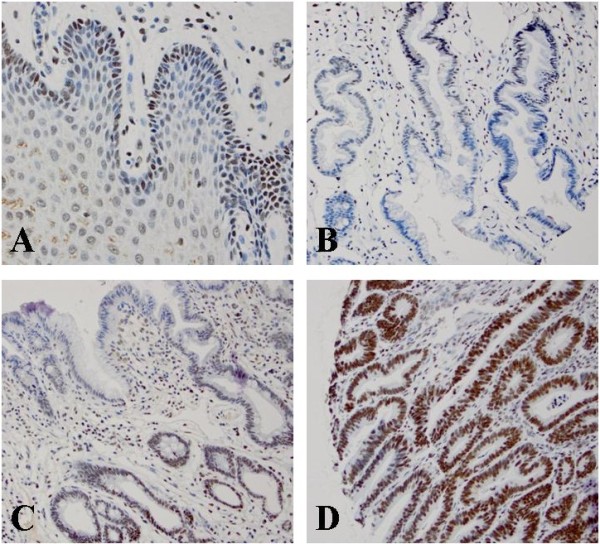
**High Bmi-1 expression in various histologic types by immunohistochemical studies. A**. Bmi-1 positive cells predominantly in the basal layer of normal esophageal squamous epithelium; **B**. Distribution of Bmi-1 positive cells mostly at the base of glands in columnar cell metaplasia; **C**. Bmi-1 positive cells mostly at the base of glands in intestinal metaplasia; **D**. Distribution of Bmi-1 positive cells evenly in the glands of low-grade dysplasia glands.

All sections were reviewed independently by BC and ZZ blinded to all clinical and pathologic information. Discordant cases were reviewed by both BC and ZZ and a final consensus was reached. The percentage (0-100%) of the cells with positive nuclear staining was recorded. The cytoplasmic staining, identified in some cases, may represent cross-reaction of anti-body. However, Bmi-1 mutation with KRMK blocks Bmi-1 nuclear translocation, which may also cause Bmi-1 staining in the cytoplasm [32]. Therefore, the cytoplasm stain was not counted in this study. The intensity of Bmi-1 nuclear staining was graded as 0, 1+, 2+, or 3+ (Figure 1). Bmi-1 protein was considered highly expressed if 10% or more of cells stained with a moderate to strong intensity (2+ and 3+, respectively).

### Statistical analysis

All the descriptive statistics in this study were presented as mean. A *P*-value of less than 0.05 was considered statistically significant. The univariate analysis of Bmi-1 was conducted first and then followed by a multivariate analysis, including age, gender, and clinical covariate: lymph node metastasis and tumor stage. We divided esophageal adenocarcinoma, high- and low- and grade dysplasia, columnar cell metaplasia as group 1, and squamous cell carcinoma and squamous epithelium as group 2. Chi-square and Fisher exact tests were used as appropriate to compare Bmi-1 positivity rate in the two groups. To evaluate the influence of amplification and high expression of Bmi-1 in esophageal adenocarcinoma and squamous cell carcinoma, comparative risk analysis using the Kaplan-Meier method compared by the log-rank test was performed with Bmi-1 amplified and non-amplified groups. All the statistical analyses were conducted with SAS 9.3 software (SAS Institute Inc., Cary, NC).

## Results

### Immunohistochemical characteristics and analysis of Bmi-1 expression

**Figure 2:**
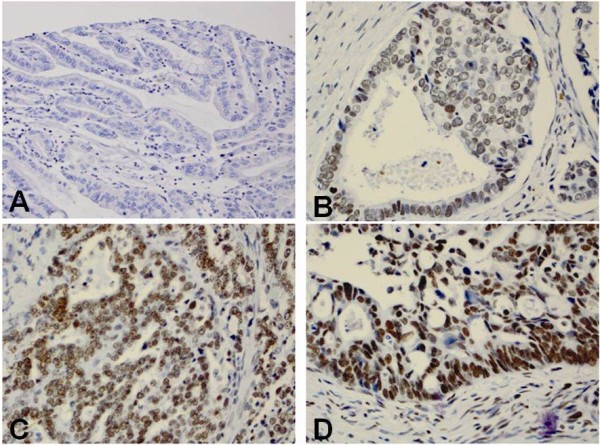
**Immunohistochemical score of Bmi-1 in esophageal adenocarcinoma (EAC). A**. No Bmi-1 expression in EAC glands (score 0); **B**. Bmi-1 weakly positive cells distributed evenly in EAC glands (score 1+); **C**. Bmi-1 moderately positive cells distributed evenly in EAC glands (score 2+); **D**. Bmi-1 strongly postive cells are distributed in mostly at the base of EAC glands (score 3+).

**Figure 3:**
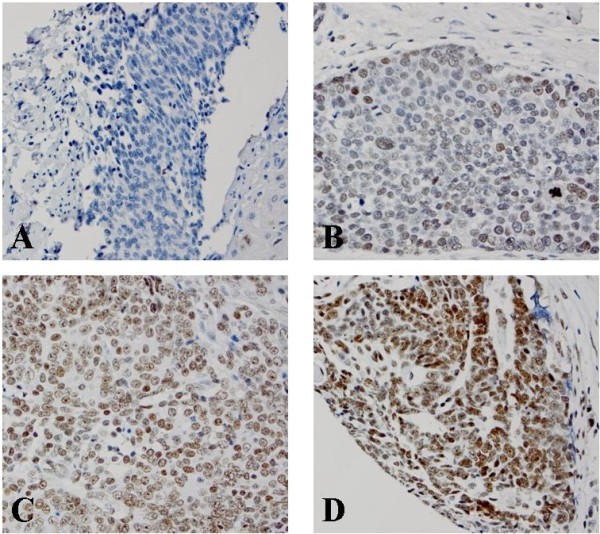
**Immunohistochemical score of Bmi-1 in esophageal squamous cell carcinoma (ESCC). A**. No Bmi-1 expression in ESCC (score 0); **B**. Bmi-1 weakly positive cells distributed evenly in ESCC (score 1+); **C**. Bmi-1 moderately positive cells distributed evenly in ESCC (score 2+); **D**. Bmi-1 strongly positive cells in ESCC (score 3+).

Bmi-1 was expressed in almost all of the esophageal specimens. The expression of Bmi-1 in normal squamous epithelium was mostly located in the basal layers, which is similar to the previous reports in other organs related to stem cells (Figure 1) [33,34]. The expression of Bmi-1 in columnar cell metaplasia and Barrett’s esophagus also distributed at the base of glands, but the intensity and percentage of Bmi-1 was greatly increased. However, the expression of Bmi-1 in low- and high-grade dysplasia, esophageal adenocarcinoma and squamous cell carcinoma was evenly distributed throughout the full lesion (Figures 1, 2 and 3).

High Bmi-1 expression was identified in all histologic types from squamous epithelium to carcinoma (Figures 1, 2 and 3). However, the percentage of high Bmi-1 expression increased following the histologic changes from squamous epithelium (7%) to columnar cell metaplasia (22%), Barrett’s esophagus (22%), low-grade dysplasia (45%), high-grade dysplasia (43%) and esophageal adenocarcinoma (37%) (Table 3). The frequency of high Bmi-1 expression in Barrett’s esophagus and columnar cell metaplasia was significantly greater than squamous epithelium (*p* < 0.05). The esophageal adenocarcinoma and low- and high-grade dysplasia groups, also showed significantly greater frequency of high Bmi-1 expression compared with the Barrett’s esophagus and columnar cell metaplasia groups (*p* < 0.05). However, there was no significant difference between esophageal adenocarcinoma, low- and high-grade dysplasia.

**Table 3:** Comparing the percentage of high Bmi-1 expression in various histologic types

**Histologic type**	**n**	**Non-high expression (%)**	**High expression (%)**
**Adenocarcinoma**	**110**	**69 (63)**	**41 (37)**
**High-grade dysplasia**	**14**	**8 (57)**	**6 (43)**
**Low-grade dysplasia**	**20**	**11 (55)**	**9 (45)**
**Barrett’s esophagus**	**36**	**28 (78)**	**8 (22)**
**Columnar cell metaplasia**	**63**	**49 (78)**	**14 (22)**
**Squamous cell carcinoma**	**34**	**25 (74)**	**9 (26)**
**Squamous epithelium**	**80**	**74 (93)**	**6 (7)**

Nine of 34 cases of esophageal squamous cell carcinoma and 6 of 80 cases of squamous epithelium showed high expression of Bmi-1 (Figure 3, Table 3). The esophageal squamous cell carcinoma group showed significantly high Bmi-1 expression compared with the squamous epithelium group (*p* = 0.008).

### Correlation of high Bmi-1 expression and clinicopathologic characteristics

The correlation of high Bmi-1 expression with clinicopathologic features was analyzed. High expression of Bmi-1 was significantly associated with poor differentiation in esophageal adenocarcinoma (67%) (Table 2). However, Bmi-1 expression was not associated with age, gender, stage, and lymph node metastasis.

### Survival analysis

**Figure 4:**
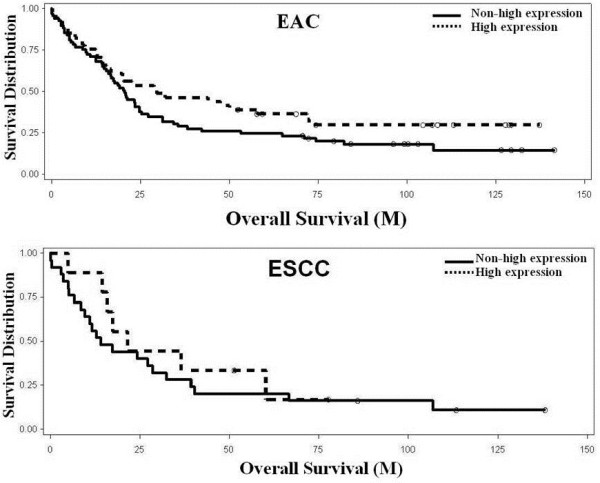
**Kaplan-Meier analysis of overall survival associated with high Bmi-1 expression and in esophageal adenocarcinoma (A) and squamous cell carcinoma (B). A**. Bmi-1 expression in EAC showed a better but not significant overall survival (*p*=0.13). **B**. Bmi-1 expression in ESCC showed no change. Solid line: non-high expression; Dotted line: high expression.

Kaplan-Meier analysis compared by the log-rank test was used to calculate the effect of the high Bmi-1 expression in patients with esophageal adenocarcinoma and squamous cell carcinoma on overall survival. For esophageal adenocarcinoma, the overall survival in the group with high Bmi-1 expression was 38.3 months, while the group with non-high Bmi-1 expression was 36.5 months. The log-rank test showed a trend towards better overall survival in the high-Bmi-1 group, but it did not reach statistical significance (*p* = 0.13, Figure 4A).

### Genomic analysis of Bmi-1 expression

**Figure 5:**
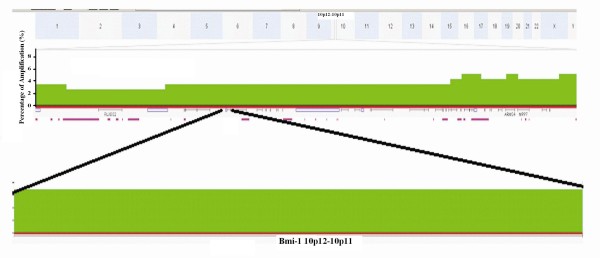
**Frequency histogram showing amplification of the Bmi-1 locus at chromosome 10p12 in 116 esophageal adenocarcinoma samples.** This locus was amplified in 4/116 (3%) cases of this patient cohort (darker green).

Analysis of 116 esophageal adenocarcinoma specimens using high density copy number microarrays revealed amplification of 3% (4/116) (Figure 5). In this cohort, the median overall survival of patients with Bmi-1 amplification was approximately 10 months and patients with no Bmi-1 amplification was 25 months. Significant association of overall survival was found with Bmi-1 amplification (*p*<0.05).

## Discussion

In this study, we reported the first evidence that high expression of Bmi-1 occurred in esophageal adenocarcinoma and its precursor lesions including, low- and high-grade dysplasia, Barrett’s esophagus and columnar cell metaplasia. High Bmi-1 expression increased in intensity and percentage following the early progress of adenocarcinoma from squamous epithelium (7%), columnar cell metaplasia (22%), Barrett’s esophagus (22%), low- (45%) and high-grade dysplasia (43%) and adenocarcinoma (37%). The increase in high Bmi-1 expression was significant from squamous mucosa to columnar cell metaplasia and Barrett’s esophagus and from Barrett’s esophagus to low-grade dysplasia and esophageal adenocarcinoma. In addition, the expression level of Bmi-1 protein was significantly associated with worse histologic grade of esophageal adenocarcinoma. However, high Bmi-1 expression did not show an association with overall survival in both esophageal adenocarcinoma and squamous cell carcinoma. DNA microarray analysis detected a low percentage of Bmi-1 amplification in esophageal adenocarciinoma.

The carcinogenesis of esophageal adenocarcinoma from normal epithelium was suggested to involve a series of events that includes the reflux of gastric and duodenal contents into the esophagus leading to reflux esophagitis, followed by Barrett’s esophagus, dysplasia, and esophageal adenocarcinoma [7]. During these events, the accumulation of genetic and epigenetic aberrations driven by inflammation, as well as acid and bile salt stimulation, contributes to the carcinogenesis. A number of genes including apoptosis-related genes, cell cycle-related genes, tumor suppressor genes, oncogenes and growth factors, have been reported to be involved in esophageal carcinogenesis. Bmi-1 has a role in epigenetic modification as a member of the polycomb-group family, and its abnormal expression may contribute to carcinogenesis [35]. We found Bmi-1 present in the basal layers of normal squamous mucosa layer where the stem cells are usually located, but the percentage of expression was very low and the intensity was weak. However, the percentage and intensity of high Bmi-1 expression were significantly increased following the progression from columnar cell metaplasia to adenocarcinoma. The percentage of high expression cells were doubled in low- and high-grade dysplasia compared with to columnar cell metaplasia and Barrett’s esophagus. Also, the distribution of cells with high Bmi-1 expression was observed to have a different pattern, mostly within the base of columnar cells metaplasia compared with the full gland distribution in dysplasia and adenocarcinoma. The trend of high Bmi-1 expression suggests that Bmi-1 may play an important role in the early carcinogenesis.

From a previous study, Bmi-1 was reported to act as a stable transcriptional repressor that regulates inhibitors of p16INK4a and p19ARF [12]. Our DNA microarray studies lends support to a previous report that p16 and p53 play important roles in early carcinogenesis of esophageal adenocarcinoma [36]. The high expression of Bmi-1 in precancerous lesion implies that Bmi-1 might promote cell proliferation by suppressing p16/Rb and/or p19ARF/MDM2/p53 tumor suppressor pathway in early carcinogenesis. Therefore, further investigation is needed for the role of Bmi-1 in relation to p16, p53, and other signal pathways.

In esophageal adenocarcinoma, the amplification of Bmi-1 was very low (3%) by DNA microarray study compared with to the high expression (37%) by immunohistochemical study. From previous studies on gastric and colonic adenocarcinoma, Bmi-1 was found to upregulate both at the transcriptional and translational levels [18,20,21]. We assumed that high Bmi-1 expression may be involved at the transcriptional level without significant DNA amplification, suggesting that the higher expression in esophageal adenocarcinoma is not driven by changes in the DNA copy number. In addition, the 16p segment, where Bmi-1 and several genes are located, is amplified. The worse overall survival may not be solid evidence to prove the association of Bmi-1 amplification with prognosis.

We then analyzed the association between Bmi-1 expression and clinical characteristics of the patients. The analysis revealed a significant correlation between high expression of Bmi-1 in esophageal adenocarcinoma with moderately to poorly differentiated esophageal adenocarcinoma. Unlike previous studies on gastric carcinoma [18], colonic [20], and esophageal squamous cell carcinoma [29], we observed no significant correlation between high expression of Bmi-1 and esophageal adenocarcinoma and squamous cell carcinoma with other clinicopathologic features such as age, gender, stage, metastatic lymph nodes. The difference in the correlation between Bmi-1 and clinicopathologic features may reflect the tissue and population variance of these studies. The previous studies were performed in the Chinese population.

While our study showed high Bmi-1 expression of shows slightly better but not a significant difference in prognosis in esophageal adenocarcinoma, most studies showed that high Bmi-1 expression was associated with poorer prognosis [18-20,24,25,27]. However, Pietersen *et al.* did report a correlation between high expression of Bmi-1 and better outcome in breast cancer patients [22]. The non-significant prognosis for esophageal adenocarcinoma was unexpected since other gastrointestinal carcinomas showed worse prognosis with high Bmi-1 expression. The different types of epithelium located throughout the gastrointestinal system, molecular mechanisms, and population groups may explain this discrepancy.

In esophageal squamous cell carcinoma, the data on the association between Bmi-1 expression and prognosis are limited and conflicting. Our results for esophageal squamous cell carcinoma are similar to the results from Yamada *et al.* where they found no association in prognosis between high expression of Bmi-1 and squamous cell carcinoma [30]. In contrast, two other reports found patients with higher expression of Bmi-1 had lower overall survival compared with patients with worse expression of Bmi-1 [28,29].

## Conclusion

In conclusion, Bmi-1 was rarely amplified in esophageal adenocarcinoma, but highly expressed in esophageal adenocarcinoma and squamous cell carcinoma. High expression of Bmi-1 was significantly correlated with the histologic grade of esophageal adenocarcinoma. The accumulation of high Bmi-1 expression from columnar cell metaplasia, Barrett’s esophagus, dysplasia to adenocarcinoma implies an important role of Bmi-1 in the early development of esophageal adenocarcinoma. It also suggests that Bmi-1 is a potential target for treatment of precancerous lesions.

## Abbreviations

Bmi-1: B cell-specific Moloney murine leukemia virus integration site 1; IHC: Immunohistochemistry; TMA: Tissue microarray; BE: Barrett’s esophagus; GERD: Gastroesophageal reflux disease.

## Competing interests

All authors declared no competing interest.

## Authors’ contribution

ZZ and JS: Designing the project; editing the paper; ZZ and BC: Scoring all IHC slides from TMA, writing the paper; YX: Involving analyzing data; TG and SB: Analyzing SNP DNA microarray data; JP, AP, DF and JL: Collecting clinicopathologic information and tissue. All authors read and approved the final manuscript.

## Pre-publication history

The pre-publication history for this paper can be accessed here:

http://www.biomedcentral.com/1471-230X/12/146/prepub
